# Antidermatophytic Activity of the Fruticose Lichen *Usnea orientalis*

**DOI:** 10.3390/medicines3030024

**Published:** 2016-09-12

**Authors:** Ashutosh Pathak, Dalip Kumar Upreti, Anupam Dikshit

**Affiliations:** 1 Biological Product Lab, Department of Botany, University of Allahabad, Allahabad 211002, India; ashupathaks@rediffmail.com; 2 Lichenology Laboratory, CSIR-National Botanical Research Institute, Rana Pratap Marg, Lucknow 226001, India; upretidknbri@gmail.com

**Keywords:** antidermatophytic, dermatophytes, DPPH, Lichen

## Abstract

In the present study, the new biological sources in the form of lichen *Usnea orientalis* Motyka was screened for its antidermatophytic potential. Six species of dermatophytes were chosen on the basis of their prevalence for antidermatophytic assays, and the Clinical Laboratory Standard Institute (CLSI)-recommended broth microdilution procedure was used to detect the efficacy of extract against dermatophytes. Thin layer chromatography of lichen extracts reveals the presence of two secondary metabolites viz. salazinic acid and usnic acid. *U. orientalis* extract exhibited promising antidermatophytic activity against all tested pathogens. Amongst all tested pathogens, *Epidermophyton floccosum* exhibited most susceptibility towards extract, whereas *Trichophyton mentagrophytes* exhibited the least susceptibility. Topical application of *U. orientalis* extract might be helpful in the cure of dermal infections.

## 1. Introduction

Lichen thallus (a composite organism) is mainly composed of mycobiont and photobiont in a mutualistic relationship [1]. Lichens are known for their secondary metabolites, which are quite unique to them and have several properties such as photoprotection, allelopathy, and antioxidant, antimicrobial, and antiviral activities [2]. The genus *Usnea* is well known for the worldwide distribution and for the role of secondary metabolite, i.e., usnic acid, in medicines. More than 300 species of genus *Usnea* were reported throughout the world, of which 57 are from India [3]. *Usnea orientalis* Motyka (fruticose and corticolous lichen) was used ethno-medicinally in urinary tract problems, swelling, and edema [4]. Cutaneous infections caused by fungi generally produce boggy nodular swelling called kerion [5]. Cutaneous mycoses (skin infection) caused by filamentous keratinophilic fungi known as dermatophytes, composed of the three genera *Trichophyton*, *Microsporum*, and *Epidermophyton* [6]. In humans, about 30 species of dermatophytes have been identified as pathogens [7]. Dermatophytes are cosmopolitan in distribution. Several reports from different parts of the world have reported the occurrence of dermatophytes. A study involving 16 European countries showed that 35%–40% of the analyzed individuals had infection of the foot (tinea pedis) caused by dermatophytes [8]. A study in the US revealed that between 22% and 55% had hair scalp infection of dermatophytes [9]. Another study conducted in Brazil showed that *Trichophyton rubrum*, *Microsporum canis*, and *Epidermophyton floccosum* were the most prevalent species infecting humans in developing countries [10]. The World Health Organization estimated that dermatophytes affect about 25% of the world population [6]. Apart from wide prevalence, the dermatophytes have exhibited resistance against griseofulvin, terbinafine, and fluconazole [6,11,12,13,14]. Although the prevalence of drug resistance in dermatophytes is rare, recurrence in patients is common with 60%–80% [5]. Based on the aforementioned literature, the new biological source in the form of *U. orientalis* was screened for its antidermatophytic property.

## 2. Material and Methods

### 2.1. Preparation and Percent Yield of Extract

Lichen thalli were collected from Koti, Chakrata district, Uttarakhand, India and identified with the help of relevant keys [15]. The voucher specimen was deposited in the Botanical Survey of India, Allahabad, India: *U. orientalis* (Accession No. BSA-8760). Two grams of air-dried thallus (vegetative as well as fruiting) was washed thoroughly using tap water followed by distilled water and pat-dried. Then, thalli were subjected to cold extraction of secondary metabolites in 50 mL of acetone. Subsequently, the solvent was filtered by Whatman No. 1 filter paper after 48 h.

The weight of crude extract obtained was 0.16 g after vacuum drying the filtrate via rotary evaporator. Percent yield of crude extract was calculated according to the equation below:

Percent yield (%) = (Dry weight of extract/Dry weight of sample) × 100.

Stock solution (50 mg/mL) of crude extract was prepared in dimethyl sulphoxide (DMSO) for the evaluation of antidermatophytic and free radical scavenging activity.

### 2.2. Thin Layer Chromatography of Extract

Solvent A (Toluene (180 mL) 1,4 dioxane (45 mL): Acetic acid (5 mL)) and Solvent C (Toluene (170 mL): Acetic acid (30 mL)) was used as mobile phase, whereas silica-coated aluminum plate (TLC Silica gel 60 F254, Merck KGaA, Darmstadt, Germany) was used as stationary phase and Usnic acid (Chemical Industry Co., Ltd., Tokyo, Japan) was taken as standard [16].

### 2.3. Test Pathogens and Inocula Preparation

Fungal cultures of *Epidermophyton floccosum* (MTCC No. 7880), *Microsporum canis* (MTCC No. 3270), *M. fulvum* (MTCC No. 7684), *M. gypseum* (MTCC No. 2867), *Trichophyton rubrum* (MTCC No. 296), and *T. mentagrophytes* (MTCC No. 7687) were procured from Microbial Type Culture Collection and Gene Bank (MTCC), Chandigarh, India, and were subcultured on Sabouraud Dextrose Agar (SDA) medium under laminar flow cabinet (Laminar flow ultra clean air unit, Micro-Filt, Pune, India). Inocula were prepared in saline media and then adjusted to a 0.5 McFarland standard, corresponding to ca 0.5 × 10^6^ CFU/mL, and transmittance of inocula prepared were 70%–72% at 520 nm for each pathogen [17].

### 2.4. Antifungal Assay for Opportunistic Filamentous Fungi

#### 2.4.1. Determination of Fungistatic Concentration

Antifungal susceptibility test was performed according to the Clinical Laboratory Standard Institute (CLSI)-recommended broth microdilution method in RPMI-1640 medium HEPES modification (Sigma Aldrich, St. Louis, MO, USA) supplemented with MOPS buffer (3-morphollinopropane-1-sulfonic acid) (Qualigens Fine Chemicals, Mumbai, India) [18]. Brief steps involved per plate were as follows: Inocula prepared was diluted 1:50 times in testing media, i.e., RPMI 1640; the test was performed in 96-well flat bottom microtiter plates; Column 1 was named as negative control consisting of 100 μL of RPMI-1640 broth media and 100 μL of inocula prepared in formaldehyde (less than 0.5%); Column 2 was named as broth control consisting of 200 μL of media; Columns 3 and 4, 6 and 7, and 9 and 10 were vertically diluted with extract having a final concentration of 1.25 to 0.009 mg/mL and named as treated; Column 5, 8, and 11 were taken as positive controls and contained only 100 μL of inocula and 100 μL of RPMI-1640 broth media, respectively; Column 12 was named as extract control and contained vertically diluted extract in the aforementioned concentrations. To nullify the effect of extract color, optical density (O.D.) of the extract control was subtracted from treated columns corresponding to extract treated [19]. Percent inhibition was calculated using following equation:

Per cent Inhibition (%) = ((O.D. positive control − O.D. extract-treated)/(O.D. positive control)) × 100.

**Figure 1:**
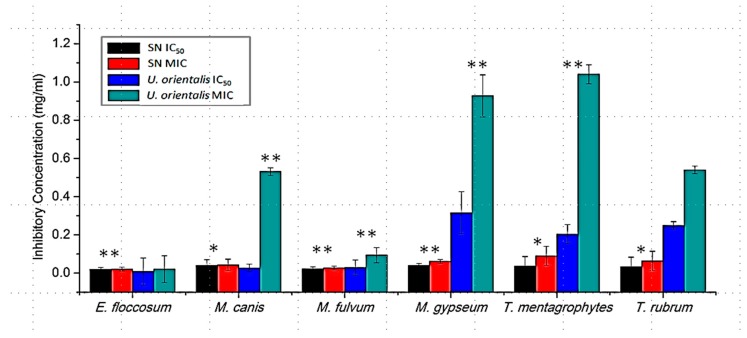
Antidermatophytic activity *U. orientalis* extract compared with Sertaconazole nitrate. Error bars show the standard error mean. ** Level of significance ≤ 0.01. * Level of significance ≤ 0.05.

Minimum inhibition concentrations (MICs) was calculated based on optical density recorded with a spectrophotometer (SpectraMax Plus^384^, Molecular Devices Corporation, Orleans Drive, Sunnyvale, CA, USA) at 530 nm after 96 h of incubation at 30 ± 2 °C (Figure 1).

The antifungal activity of the chemical drug Sertaconazole nitrateBP (SN) (Glenmark Pharmaceuticals, Nasik, India) was taken as the reference standard. An amount of 50 mg/mL of stock solution of SN was prepared and tested at the same concentrations as the *U. orientalis* extract.

#### 2.4.2. Determination of Fungicidal Concentration

A portion of 20 µL of *U. orientalis-*treated columns from the above MIC wells were transferred into 7-mL tubes of a fresh RPMI 1640 medium. Tubes were incubated at 30 ± 2 °C for 4 weeks and checked for the turbidity. The aforementioned procedure was performed with SN, and the concentration at which no turbidity has been achieved was defined as the minimum fungicidal concentration (MFC) [20,21].

### 2.5. Statistical Analysis

An independent sample *t*-test was performed between the positive control and sertacoazole-treated dermatophytes; and between the positive control and extract-treated dermatophytes for the measure of Levene’s test for equality of variances and *t*-test for equality of means via SPSS v20.

## 3. Results and Discussion

### 3.1. Percent Yield of Extract

Percent yield of extract obtained from *U. orientalis* thallus was 8%.

### 3.2. Thin Layer Chromatography

A light yellow turning into a green-colored spot was observed and confirmed as usnic acid in *U. orientalis* extract and the other compound was salazinic acid. The chromatographic study confirmed the presence of two compounds viz. usnic acid and salazinic acid in the *U. orientalis* extract.

### 3.3. Antifungal Test for Opportunistic Filamentous Fungi

*U. orientalis*, fruticose lichen, contains usnic and salazinic acid and is used ethno-medicinally in swelling. The *U. orientalis* extract was tested for its efficacy against six dermatophytic species and compared with sertaconazole nitrate. Sertaconazole nitrate is a highly active chemical drug having low fungistatic and fungicidal activities, but it causes inflammation and itching in patients [22]. Due to the side effects of SN and the development of resistance in dermatophytes against first-line clinical drugs, there is a need for new antidermatophytic compounds. The antidermatophytic activity of SN and *U. orientalis* were represented graphically in the form of IC_50_ (50% inhibitory concentration) and MIC (minimum inhibitory concentration) values were represented graphically in Figure 1.

*E. floccosum* exhibited equal susceptibility towards SN and *U. orientalis* extract with an MIC value equivalent to 0.021 mg/mL and an MFC value equivalent to 0.039 mg/mL. The IC_50_ value for *E. floccosum* was achieved at 0.009 mg/mL against *U. orientalis* and 0.020 mg/mL against SN. *M. gypseum* was found least susceptible among *Microsporum* spp. with IC_50_ = 0.040 mg/mL; MIC = 0.062 mg/mL; MFC = 0.078 mg/mL against SN and IC_50_ = 0.316 mg/mL; MIC = 0.927 mg/mL; MFC = 1.250 mg/mL against *U. orientalis*. *M. fulvum* was found most susceptible among *Microsporum* spp. with IC_50_ = 0.022 mg/mL; MIC = 0.027 mg/mL; MFC = 0.039 mg/mL against SN and IC_50_ = 0.029 mg/mL; MIC = 0.094 mg/mL; MFC = 0.156 mg/mL against *U. orientalis*. The IC_50_, MIC, and MFC for *M. canis* were obtained at 0.040 mg/mL, 0.043 mg/mL, and 0.078 mg/mL for SN and 0.027 mg/mL, 0.531 mg/mL, and 0.625 mg/mL for *U. orientalis* extract.

The IC_50_, MIC, and MFC for *T. rubrum* were achieved at 0.033 mg/mL, 0.064 mg/mL, and 0.078 mg/mL against SN and 0.249 mg/mL, 0.54 mg/mL, and 0.625 mg/mL against *U. orientalis* extract. *T. mentagrophytes* was found least susceptible amongst all tested pathogens with IC_50_ = 0.037 mg/mL, MIC = 0.09 mg/mL, and MFC = 0.156 mg/mL against SN and IC_50_ = 0.204 mg/mL, MIC = 1.04 mg/mL, and MFC = 1.25 mg/mL against *U. orientalis* extract. The efficacy of *U. orientalis* extract is equivalent to sertaconazole nitrate against *E. floccosum*, but was found to be less effective against all other dermatophytes.

In another study, extracts of *U. florida* exhibited an MIC between 0.050 and 0.100 mg/mL against *M. gypseum*, *T. mentagrophytes*, and *T. rubrum* [23]. In the present study *U. orientalis* exhibited a MIC between 0.531 and 1.04 mg/mL and was found to be less active than *U. florida*. The ethno-medicinal use of *U. orientalis* extract in swelling caused by dermatophyte infection might be cured, but in vivo efficacy and potency of the lichen extract needs to be investigated.

### 3.4. Statistical Analysis

The level of significance was calculated in terms of *p*-value. Results obtained were statistically significant except between *U. orientalis* treatments and control *T. rubrum* (*p*-value = 0.19) and *U. orientalis* treatments and control *E. floccosum* (*p*-value = 0.19); *p*-values calculated for SN-treated *E. floccosum*, *M. fulvum*, and *M. gypseum* were less than 0.01, whereas, for *M. canis*, *T. mentagrophytes*, and *T. rubrum*, *p*-values were less than 0.05. *p*-value calculated for *U. orientalis* treated all pathogens except *T. rubrum, which* showed a level of significance less than 0.01 (Figure 1).

## 4. Conclusions

*U. orientalis* extract exhibited broad-range antidermatophytic activity against all three genera of dermatophytes, and usnic acid (a well-known antifungal compound) was present in the lichen extract. Topical application of lichen extract might be helpful in the cure of cutaneous infections.
